# Supplementary material for: The conserved microRNA miR-34 regulates synaptogenesis via coordination of distinct mechanisms in presynaptic and postsynaptic cells
Source: Nat Commun. 2020 Feb 27;11:1092. doi: 10.1038/s41467-020-14761-8 (PMC7046720; doi:10.1038/s41467-020-14761-8)
Supplement: Supplementary file 1 — Supplementary Information [file 41467_2020_14761_MOESM1_ESM.pdf]

Supplementary Figures

The Conserved microRNA miR-34 Regulates Synaptogenesis via Coordination of Distinct Mechanisms in Presynaptic and Postsynaptic Cells

McNeill et al.

a

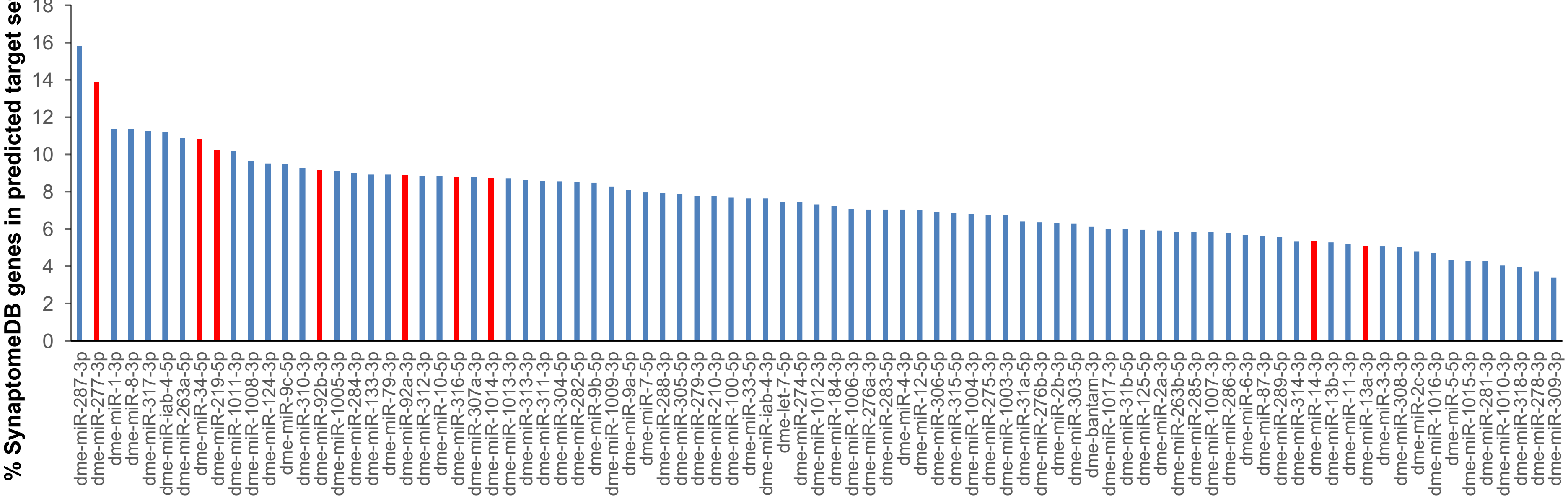

b

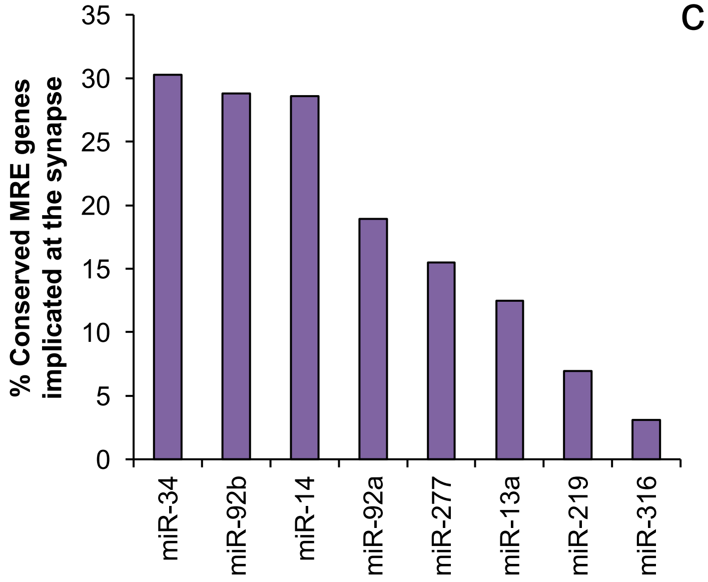

c

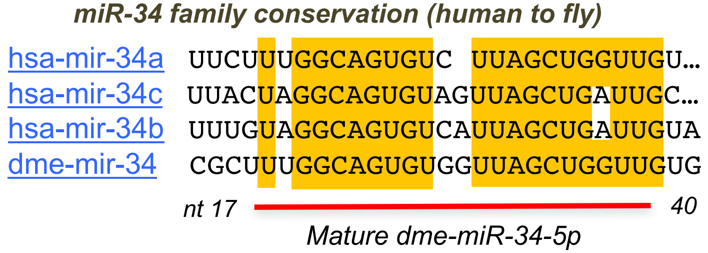

Ranking and Conservation of miRNAs in Synaptogenesis.

(a) Percentage of Synaptome DB genes known to play a role at the synapse which are also predicted to be direct targets of individual microRNAs. Hits from the NMJ morphology screen are indicated as red bars and are found across the distribution for the 87 microRNAs. Note, at the time of analysis, miR-973 which was a hit in the morphology screen was not in the data bases for target prediction, but was analyzed using the same databases at a later date (1/25/2016). At this time 9.96% of predicted targets were found in Synaptome DB which would place it between miR-1011-3p and miR-1008-3p on the chart. (b) miRNAs organized in order of those with the highest percentage of conserved target genes which have been implicated in previous *Drosophila* screens at the NMJ. (c) Conservation of mature miR-34-5p sequence among human (hsa) miR-34 a, b, and c with *Drosophila* (dme) miR-34-5p. Identical conserved nucleotides are indicated in yellow highlight.

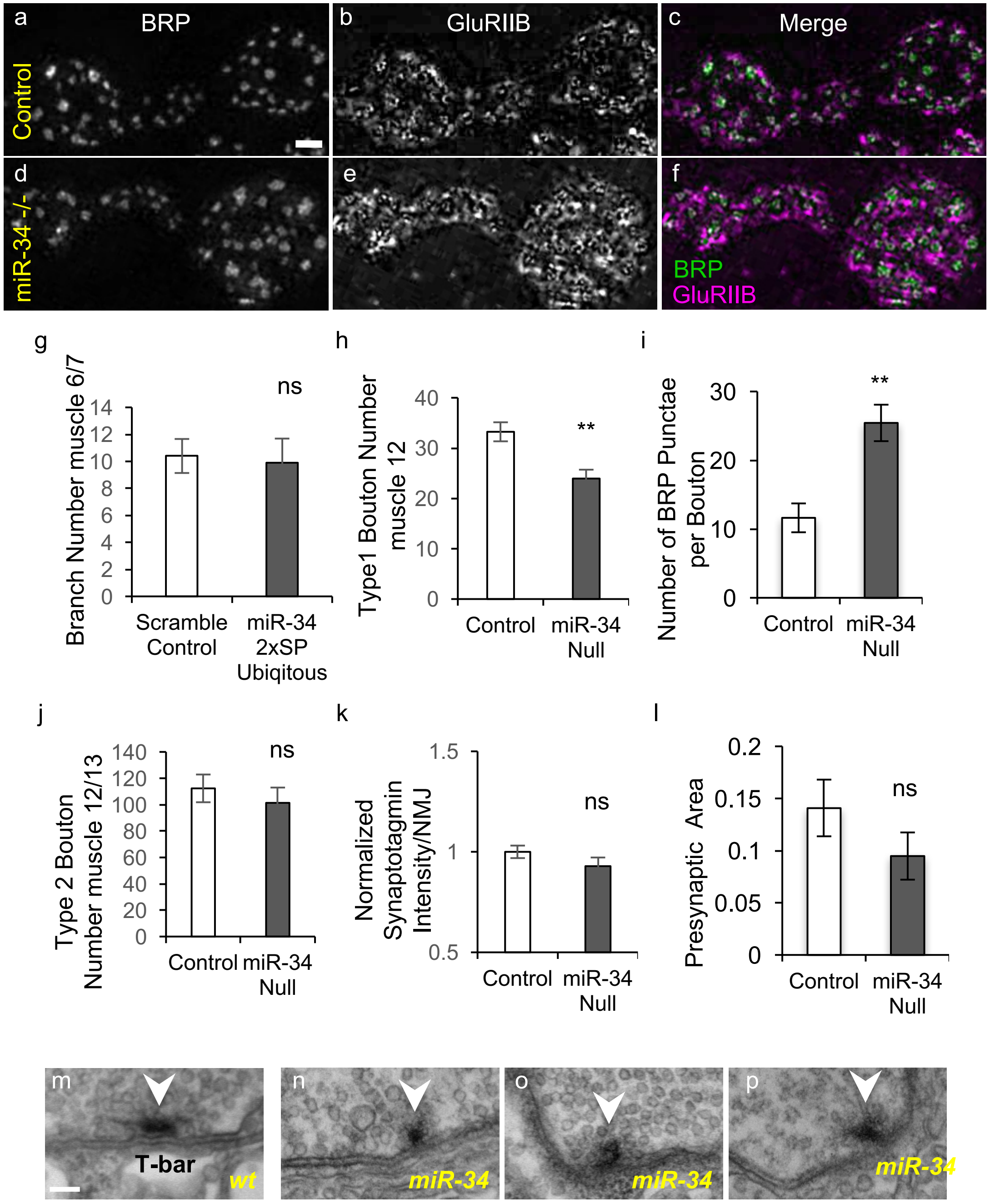

Analysis of miR-34 Null Pre- and Post-synaptic Phenotypes.

(a-c) Control NMJs imaged with 3-D SIM reveal normal AZ puncta with Brp (white in a; green in c merge) and GluRIIB (white in b; magenta in c merge) at type 1 boutons . (d-f) *miR-34* nulls display altered size, distribution and intensity of Brp (white in d; green in f merge) and GluRIIB (white in e; magenta in f merge). Scale bar is 5 nm.

(g) Number of branches in muscle 6/7 NMJ in segment A2 from arbors labeled with HRP. No significant difference (ns) was observed (n=12 L3 larvae from each genotype). (h) Type 1 bouton number in NMJ arbor spanning muscle 12/13. ** p-value $\leq$ 0.01 (n=12 L3 larvae from each genotype). (i) Number of BRP punctae per bouton based on OMX images of NMJ from muscle 6/7 segment A2 co-stained with HRP ** p-value $\leq$ 0.01 n=4 animals from each genotype. (j) Type 2 bouton number in NMJ arbor spanning M12 no significant difference was observed between the two groups (n=12 L3 larvae from each genotype). (k) Intensity quantification of synaptic Synaptotagmin at the M6/7NMJ relative to HRP and normalized to control; no significant difference observed (ns, n=12 L3 larvae from each genotype). (l) Quantification of presynaptic area from TEM images (ns) n= 7 *miR-34* null animals, n=3 w^{1118} animals, ~20 bouton images per animal). (m-p) T-bar structure in TEM images (m) wild-type (w^{1118}) and (n-p) *miR-34* null, an array of observed phenotypes demonstrating lack of typical wild type organization. T-bar indicated by white arrowhead (Scale bar: 100 nm).

Supplementary Fig. 3

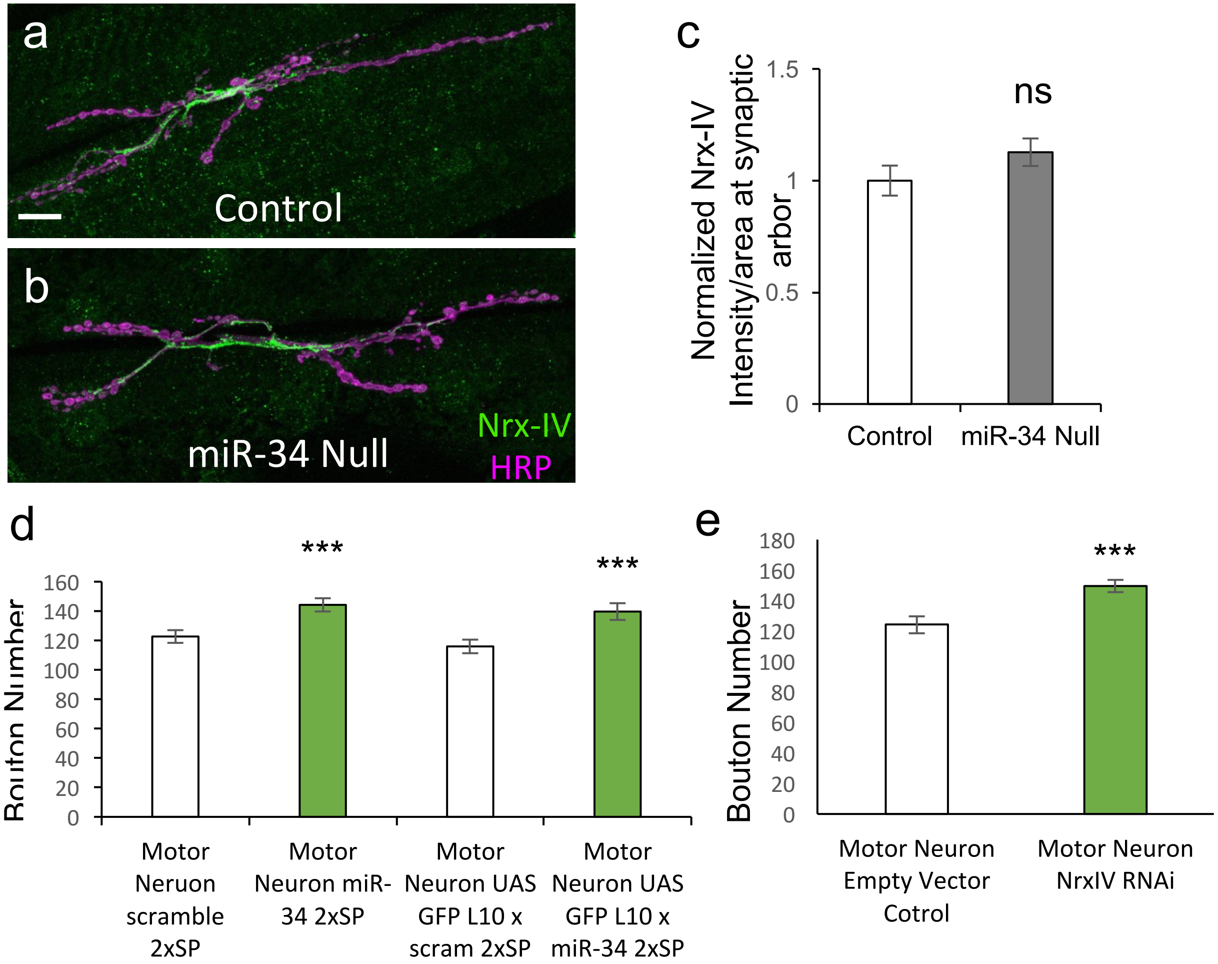

Analysis of Nrx-IV Expression and Function.

(a, b) Nrx-IV antibody staining along the central M6/7NMJ synaptic arbor confirms published observations that the majority of peripheral Nrx-IV accumulates in the glia (green) that ensheath motor axons before they extend and branch over the muscle surface to elaborate presynaptic boutons visualized with anti-HRP (magenta). A normal pattern of staining in the glial processes typical of control (*w^{1118}*, a) appears unchanged in the *miR-34* null (b, scale bar: 10 microns). (c) Overall intensity of Nrx-IV relative to HRP intensity is not significantly (ns) different from control in *miR-34* null animals (c, segment 2, muscle 6/7 n=12 L3 larvae from each genotype). (d) As a control experiment to determine if adding a second UAS-transgene into the *miR-34SP* background might dilute the effect of the inhibition due to dilution of GAL4 activity, we compared the presynaptic phenotype of *miR-34SP* under control of *OK6-GAL4* to a combination of *miR-34SP* with *UAS-GFP-L10a*. Both of these *miR-34SP* genotypes showed a significant increase in type 1 boutons at the M6/7NMJ compared to their respective matched *ScrambleSP* controls (*** represents $p \leq 0.001$; Student's t-test, d, segment 2, muscle 6/7 n=20 L3 larvae from each genotype). There was no significant difference between the two miR-34SP inhibition genotypes ($p \leq 0.53$). (e) Motor neuron-specific RNAi knock down of Nrx-IV displays a significant increase in type 1 bouton number compared to an empty vector control strain (*** represents $p \leq 0.001$) segment 2, muscle 6/7; n=20 L3 larvae from each genotype.

Supplementary Fig. 4

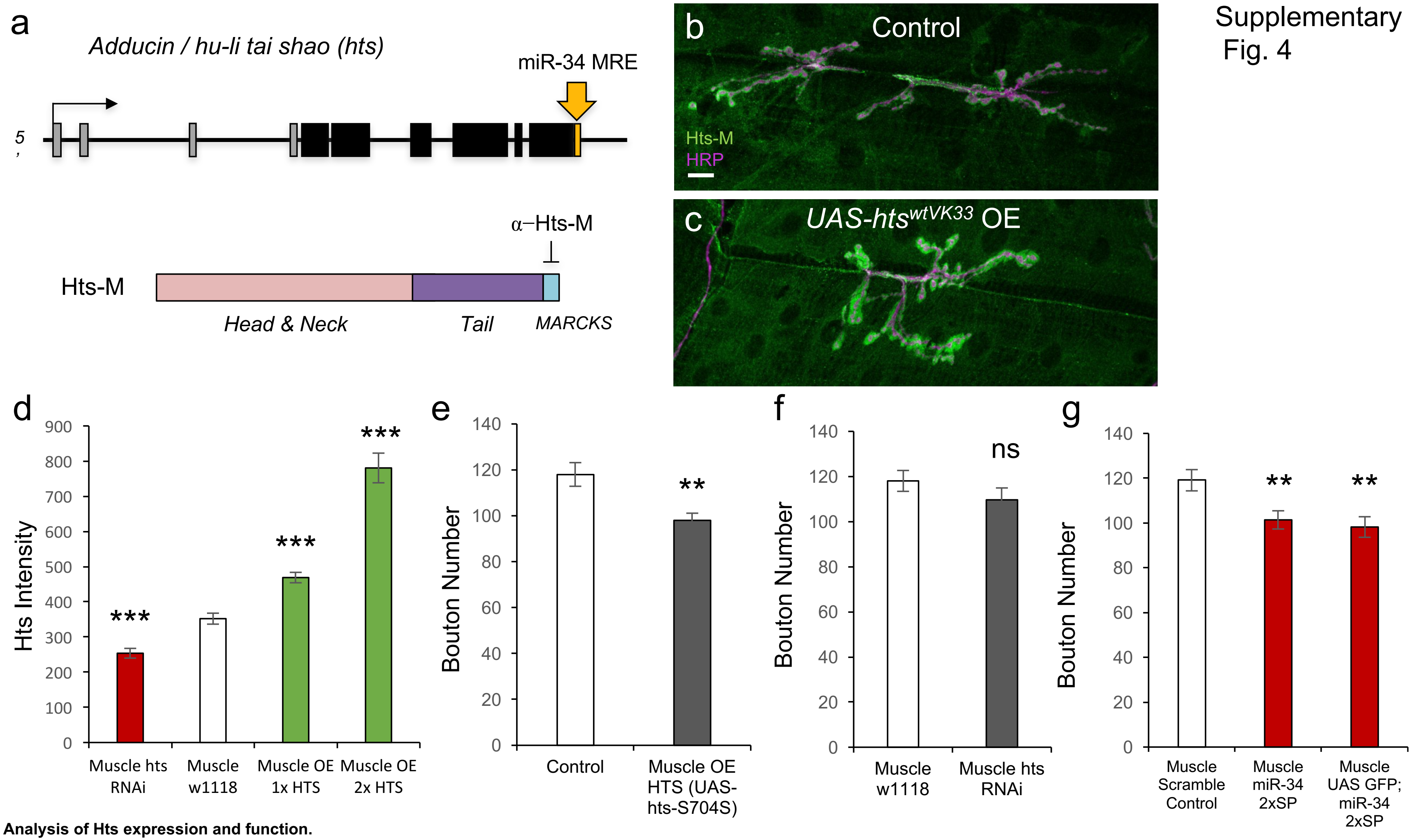

Analysis of Hts expression and function.

(a) Schematic of the *Drosophila hts* locus, location of the miR-34 MRE is indicated in gold. The Hts-M isoform present at the *Drosophila* NMJ encodes a 718aa long protein that contains a C-terminal MARCKS peptide. The peptide epitope of the Hts-M antibody is indicated. (b,c) Antibody to Hts-M protein isoforms (green) surrounding presynaptic boutons stained with anti-HRP (magenta) in *w^{1118};DMef-GAL4* control (b) or single transgene expression with *DMef-GAL4;UAS-hts-M^{wtVK33}* in (c) (Scale bar: 10 microns). (d) Using a new affinity-purified anti-Hts-M antibody (see Methods), we performed a quantitative intensity analysis of muscle staining in a range of genotypes where *DMef2-GAL4* was used to drive *UAS-hts^{RNAi}, 1x UAS-hts-M^{wtVK33}*, *2x UAS-hts-$M^{wtVK33,wtZH51C}$* compared to *DMef2-GAL4* crossed with *w^{1118}*. Hts-M was significantly reduced by knock down, and showed a significant dose-dependent increase relative to the *w^{1118};DMef2-GAL4* control (*** represents p≤0.001); n=20 NMJs per genotype except for *2xUAS-hts-$M^{wtVK33,wtZH51C}$* (Muscle hts gof 2x, n=16 NMJs). (e) Type 1 bouton number in *1x UAS-hts^{s704s}* indicates a significant decrease in bouton number with Hts OE relative to *DMef2-GAL4* crossed with *w^{1118}* control (** p-value ≤ 0.01; n=20 for each genotype). (f) To test whether muscle-specific knock-down of Hts expression using *UAS-hts^{RNAi}* might have an impact on type 1 bouton number at the M6/7NMJ, we expressed this transgene with *DMef2-GAL4* and compared the phenotype to *w^{1118};DMef2-GAL4* and found no significant decrease *(n=20* NMJs in both). (g) As a control experiment to determine if adding a second UAS-transgene into the *miR-34SP* background might dilute the effect of the inhibition due to dilution of GAL4 activity, we compared the postsynaptic phenotype of *miR-34SP* under control of *DMef2-GAL4* to a combination of *miR-34*SP with *UAS-GFP*. Both of these *miR-34SP* genotypes (red bars) showed a significant decrease in type 1 boutons at the M6/7NMJ compared to matched *ScrambleSP* control (** represents p≤0.01; Student's t-test; segment A2, muscle 6/7; n=20 L3 larvae from *DMef2-GAL4* crossed with *ScrambleSP* and *miR-34SP*, n=18 *DMef2-GAL4 crossed with UAS GFP*;*miR-34SP*). There was no significant difference between the two *miR-34SP* inhibition genotypes (p≤0.6).

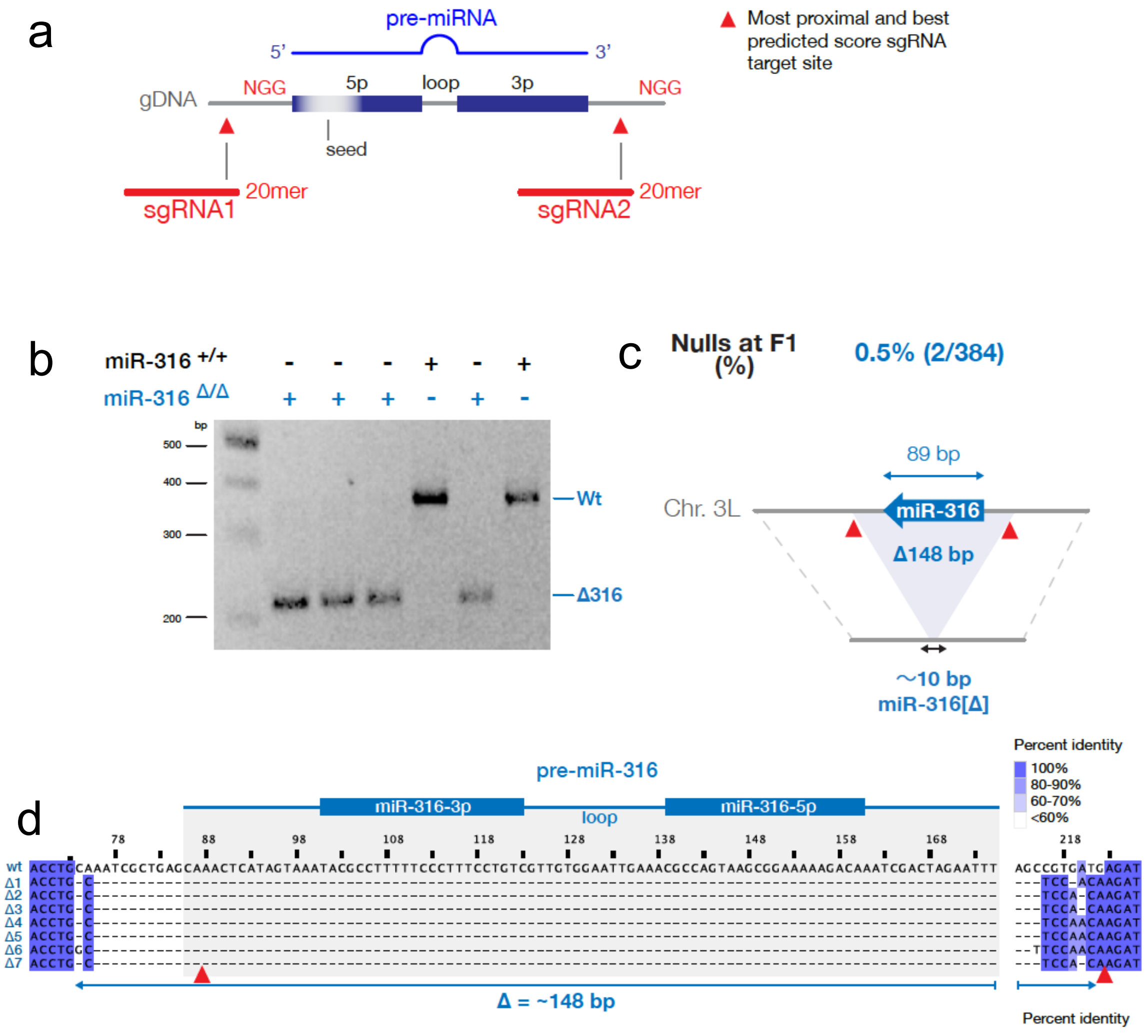

Design of miR-316 null mutant by CRISPR.

(a) Two flanking small guide RNA sequences (sgRNA) to the miR-316 locus were selected through an algorithm considering the highest stringency possible (http://www.flyrnai.org/crispr2/). Selected candidates were cloned and fused to a chimeric RNA (ChRNA) backbone required for proper loading into Cas9, under the control of an RNAPOl III promoter (U6), using the core facility, (http://gepg.hms.harvard.edu/GEPG-SRC.html). Vectors carrying a single sgRNA sequence per plasmid were then co-injected in pairs into fly embryos constitutively expressing Cas9 nuclease (Bestgene, Chino Hills, CA). Genotyping of G0 individuals was completed using PCR from genomic DNA (gDNA), primers were designed to flank the locus of interest. Products were analyzed by gel electrophoresis to identify null mutants (b). We observed a 0.5% rate of success based on our screening of 384 F1 males (c). Sequencing was completed and a 148 bp deletion including the region for miR-316 was observed (d). Selected individuals were outcrossed to w1118 and balanced with genetic markers to stabilize the new KO line.
