# Supplementary material for: The conserved microRNA miR-34 regulates synaptogenesis via coordination of distinct mechanisms in presynaptic and postsynaptic cells
Source: Nat Commun. 2020 Feb 27;11:1092. doi: 10.1038/s41467-020-14761-8 (PMC7046720; doi:10.1038/s41467-020-14761-8)
Supplement: Supplementary file 4 — Description of Additional Supplementary Files [file 41467_2020_14761_MOESM4_ESM.pdf]

Description of Additional Supplementary Files

File Name: Supplementary Data 1
Description: Primary NMJ Morphology Screen of microRNA SPonge Lines.

Mean type 1 bouton counts are shown for all 131 *miR-SP* lines in our primary screen with *tubulin-GAL4*. Standard deviation (std) and Standard Error of the Mean (sem) values are shown for each line, as is a p value for the comparison with *tubulin-GAL4;Scramble-SP control* (calculated with ANOVA). Sampling depth was 10 hemisegments per animal; due to batch processing limitations, genotypes were processed in sets of 10-12 samples, and each set included a *Scramble-SP* control.

File Name: Supplementary Data 2
Description: A List of *Drosophila* Genes Known to Display Function(s) at the Neuromuscular Junction.

Supplementary Data 2 provides a list of 470 loci (listed by both Flybase FBGN ID and gene sy mbol) that have been identified in one of several previous genetic screens for abnormalities in NMJ form and/or function; the studies are cited in the text. This list includes both overexpression and loss-of-function data. See text for data source references.
