# Supplementary material for: The conserved microRNA miR-34 regulates synaptogenesis via coordination of distinct mechanisms in presynaptic and postsynaptic cells
Source: Nat Commun. 2020 Feb 27;11:1092. doi: 10.1038/s41467-020-14761-8 (PMC7046720; doi:10.1038/s41467-020-14761-8)
Supplement: Supplementary file 5 — Supplementary Data 1 [file 41467_2020_14761_MOESM5_ESM.pdf]

Supplementary Data 1

miR-SP	Bouton # avg	std	sem	p-value
let-7SP	114.75	31.74	9.16	0.38
mir-1SP	141.30	30.52	9.65	0.27
mir-2aSP	131.92	25.47	8.05	0.34
mir-2bSP	95.44	21.25	7.08	0.68
mir-2cSP	114.80	31.70	10.02	0.61
mir-3SP	114.60	13.91	4.40	0.52
mir-4SP	88.00	36.18	11.44	0.41
mir-5SP	91.27	38.30	11.55	0.08
mir-6SP	133.40	34.14	10.80	0.55
mir-7SP	86.75	37.69	11.92	0.14
mir-9bSP	116.50	24.69	8.73	0.90
mir-9cSP	87.44	22.32	7.44	0.37
mir-10SP	117.70	54.62	17.27	0.80
mir-11SP	100.40	19.59	6.20	0.12
mir-12SP	115.70	28.97	9.16	0.63
mir-13aSP	86.70	35.84	11.33	0.04
mir-13bSP	111.40	19.18	6.06	0.41
mir-14SP	80.71	17.52	5.54	0.00
mir-31aSP	90.42	50.26	19.00	0.23
mir-31bSP	106.50	21.08	6.67	0.25
mir-33SP	118.10	20.78	6.57	0.72
mir-34SP	82.64	28.71	8.66	0.01
mir-79SP	98.63	42.36	14.98	0.85
mir-87SP	112.63	23.75	7.51	0.52
mir-92aSP	83.70	15.52	4.91	0.01
mir-92bSP	65.71	24.32	7.69	0.00
mir-100SP	115.40	30.66	9.70	0.63
mir-125SP	114.70	27.71	8.76	0.59
mir-133SP	122.91	32.07	9.67	0.99
mir-184SP	133.09	27.09	8.17	0.05
mir-190SP	66.00	19.64	6.21	0.00
mir-193SP	105.79	19.98	6.32	0.29
mir-210SP	136.00	32.48	10.27	0.09
mir-219SP	94.53	15.86	4.78	0.02

mir-252SP	139.00	42.87	12.93	0.39
mir-263aSP	99.80	49.60	15.68	0.26
mir-263bSP	152.67	46.25	15.42	0.15
mir-274SP	148.20	34.65	10.96	0.15
mir-275SP	114.33	51.56	19.49	0.49
mir-276*SP	107.70	11.74	3.71	0.25
mir-276aSP	103.33	31.22	9.87	0.15
mir-276bSP	106.30	25.06	7.56	0.30
mir-277SP	157.09	46.95	14.15	0.01
mir-278SP	88.15	12.95	3.59	0.29
mir-279SP	70.10	18.60	5.88	0.00
mir-281SP	96.50	20.17	7.13	0.08
mir-281-1*SP	114.00	26.69	7.71	0.53
mir-281-2*SP	85.50	39.22	13.07	0.09
mir-282SP	120.36	29.24	8.82	0.85
mir-283SP	117.00	27.30	8.63	0.69
mir-284SP	98.00	21.48	6.79	0.09
mir-285SP	102.61	30.24	6.31	0.11
mir-286SP	109.46	32.79	10.37	0.62
mir-287SP	80.29	20.59	7.78	0.02
mir-288SP	99.33	35.80	10.33	0.13
mir-289SP	139.00	26.42	8.36	0.31
mir-303SP	99.22	38.71	12.90	0.11
mir-304SP	166.18	32.65	9.84	0.01
mir-305SP	128.78	25.55	8.08	0.72
mir-306SP	128.00	46.83	12.09	0.13
mir-307SP	144.70	36.69	11.60	0.22
mir-308SP	151.27	19.42	5.85	0.05
mir-309SP	142.09	34.16	10.30	0.26
mir-315SP	128.40	27.94	8.84	0.74
mir-316SP	169.90	29.68	9.38	0.01
mir-317SP	147.00	26.00	8.22	0.13
mir-318SP	108.20	46.59	14.73	0.75
mir-375SP	97.10	37.41	11.83	0.15
mir-927SP	135.70	33.27	10.52	0.45
mir-932SP	83.91	24.55	7.40	0.02
mir-954SP	105.22	36.14	11.43	0.20

mir-955SP	126.36	35.72	10.77	0.85
mir-956SP	107.58	27.92	8.06	0.29
mir-957SP	143.90	32.36	10.23	0.22
mir-958SP	140.20	42.26	13.36	0.37
mir-959SP	122.00	25.66	8.12	0.94
mir-960SP	113.09	34.16	10.30	0.50
mir-961SP	104.00	22.15	7.00	0.52
mir-962SP	118.07	29.41	8.87	0.86
mir-963SP	105.50	29.61	9.36	0.26
mir-964SP	118.08	47.16	13.61	0.79
mir-965SP	115.12	38.65	9.37	0.35
mir-966SP	137.20	40.22	12.72	0.08
mir-967SP	107.00	36.13	14.75	0.73
mir-968SP	124.08	38.92	11.23	0.21
mir-969SP	143.70	49.11	14.81	0.33
mir-970SP	151.40	32.06	10.14	0.01
mir-971SP	134.33	31.42	10.47	0.18
mir-972SP	98.33	45.36	18.52	0.99
mir-973SP	98.45	22.51	6.79	0.03
mir-974SP	73.90	22.06	6.98	0.19
mir-975SP	121.64	17.70	5.34	0.18
mir-976SP	124.36	31.46	9.48	0.18
mir-977SP	106.20	15.87	5.02	0.66
mir-978SP	88.94	22.69	5.35	0.01
mir-979SP	181.80	65.63	20.75	0.01
mir-980SP	135.90	45.89	14.51	0.11
mir-981SP	128.30	27.46	8.68	0.13
mir-982SP	92.77	30.64	9.24	0.10
mir-983SP	87.30	24.12	7.63	0.55
mir-984SP	111.60	28.44	8.99	0.50
mir-985SP	124.45	49.51	14.93	0.26
mir-986SP	87.16	31.25	7.17	0.01
mir-987SP	111.33	29.85	9.95	0.53
mir-988SP	108.55	42.99	13.60	0.64
mir-989SP	88.33	33.35	11.12	0.06
mir-990SP	110.10	25.16	7.96	0.39
mir-991SP	110.00	33.60	10.62	0.43
mir-992SP	106.20	36.12	11.42	0.57

mir-994SP	126.10	27.52	8.70	0.85
mir-996SP	102.15	22.69	6.29	0.11
mir-997SP	97.37	35.86	6.90	0.07
mir-998SP	108.60	30.59	9.67	0.74
mir-999SP	84.07	39.65	11.95	0.03
mir-1001SP	106.20	22.41	7.09	0.23
mir-1002SP	103.00	36.07	11.41	0.25
mir-1003SP	115.10	24.08	7.61	0.59
mir-1004SP	102.78	49.88	17.64	0.67
mir-1005SP	103.09	21.79	6.89	0.50
mir-1006SP	123.80	28.63	9.05	0.20
mir-1007SP	115.00	23.38	7.05	0.36
mir-1008SP	112.50	35.46	11.21	0.49
mir-1009SP	127.09	25.76	7.77	0.13
mir-1010SP	100.14	45.23	18.46	0.71
mir-1011SP	95.75	23.92	6.91	0.08
mir-1012SP	127.50	37.67	11.91	0.17
mir-1013SP	118.42	64.56	18.64	0.44
mir-1014SP	93.90	18.67	5.90	0.05
mir-1015SP	116.78	50.54	17.87	0.63
mir-1016SP	128.17	54.14	22.10	0.30
mir-1017SP	100.20	43.07	13.62	0.17
Scramble Control	122.40	34.70	10.97	
