# Supplementary material for: The conserved microRNA miR-34 regulates synaptogenesis via coordination of distinct mechanisms in presynaptic and postsynaptic cells
Source: Nat Commun. 2020 Feb 27;11:1092. doi: 10.1038/s41467-020-14761-8 (PMC7046720; doi:10.1038/s41467-020-14761-8)
Supplement: Supplementary file 6 — Supplementary Data 2 [file 41467_2020_14761_MOESM6_ESM.pdf]

Supplementary Data 2

FBGN ID	Symbol
FBgn0038659	EndoA
FBgn0002973	numb
FBgn0017550	Rga
FBgn0020497	emb
FBgn0030000	CG2260
FBgn0034025	Pgant1
FBgn0031020	Naa15-16
FBgn0262743	Fs(2)Ket
FBgn0031681	Pgant5
FBgn0036059	nudE
FBgn0037534	ELOVL
FBgn0026160	tna
FBgn0261041	stj
FBgn0266672	Sec8
FBgn0003205	Ras85D
FBgn0043550	Tsp68C
FBgn0020412	JIL-1
FBgn0028509	CenG1A
FBgn0260960	Baldspot
FBgn0029996	UbcE2H
FBgn0029161	slmo
FBgn0266410	CG45050
FBgn0086710	RpL30
FBgn0035400	CG11537
FBgn0024182	waw
FBgn0267849	Syx7
FBgn0030336	CG1578
FBgn0024222	IKKbeta
FBgn0026597	Axn
FBgn0261394	Prosalpha3
FBgn0036514	CG12301
FBgn0004885	tok
FBgn0030276	Dlic
FBgn0263351	AP-2mu
FBgn0004880	scrt
FBgn0031266	Sf3b1

FBgn0046704	Liprin-alpha
FBgn0286213	RpS12
FBgn0038678	mRpL55
FBgn0004888	Scsalpha1
FBgn0034368	zda
FBgn0263995	cpo
FBgn0027280	l(1)G0193
FBgn0040230	dbo
FBgn0002787	Rpn8
FBgn0260936	scny
FBgn0031990	PAPLA1
FBgn0061492	loj
FBgn0003301	rut
FBgn0023172	RhoGEF2
FBgn0033661	CG13185
FBgn0002645	Map205
FBgn0261383	IntS6
FBgn0264959	Src42A
FBgn0052262	CG32262
FBgn0035851	MED24
FBgn0285925	Fas1
FBgn0010235	Klc
FBgn0044412	Scim15
FBgn0036686	CG7728
FBgn0039300	RpS27
FBgn0259174	Nedd4
FBgn0026760	Tehao
FBgn0086698	frtz
FBgn0037328	RpL35A
FBgn0030241	feo
FBgn0026713	l(1)G0007
FBgn0003459	stwl
FBgn0003310	S
FBgn0037529	mRpS9
FBgn0004436	Ubc6
FBgn0261792	snRNP-U1-C
FBgn0038499	Brf
FBgn0030964	Pvf1
FBgn0010382	CycE

FBgn0039009	CG13842
FBgn0026149	BCL7-like
FBgn0031030	Tao
FBgn0000547	ed
FBgn0039858	CycG
FBgn0263351	AP-2mu
FBgn0086134	Prosalpha2
FBgn0040493	grsm
FBgn0011672	Mvl
FBgn0014469	Cyp4e2
FBgn0031951	r2d2
FBgn0029879	APC7
FBgn0266671	Sec6
FBgn0023175	Prosalpha7
FBgn0015929	dpa
FBgn0028554	x16
FBgn0005642	wdn
FBgn0016977	spen
FBgn0031850	Tsp
FBgn0038185	CG14363
FBgn0038294	Mf
FBgn0036980	RhoBTB
FBgn0003862	trx
FBgn0041164	armi
FBgn0027783	SMC2
FBgn0086676	spin
FBgn0011655	Med
FBgn0041180	Tep4
FBgn0040227	eIF3d1
FBgn0028692	Rpn2
FBgn0034503	MED8
FBgn0036815	HipHop
FBgn0015544	spag
FBgn0003654	sw
FBgn0003261	Rm62
FBgn0000139	ash2
FBgn0040309	Jafrac1
FBgn0260945	Atg1
FBgn0030306	Spase25

FBgn0026316	Ubc10
FBgn0013765	cnn
FBgn0039640	CG14516
FBgn0005278	Sam-S
FBgn0027364	Six4
FBgn0036340	SRm160
FBgn0031126	Cyp6v1
FBgn0263102	psq
FBgn0028577	hfp
FBgn0036928	Tom20
FBgn0286567	gry
FBgn0028371	jbug
FBgn0032884	Pomp
FBgn0037632	CCT7
FBgn0020224	Cbl
FBgn0028402	Sin
FBgn0024734	PRL-1
FBgn0003996	w
FBgn0003701	thr
FBgn0010379	Akt1
FBgn0285963	CG46339
FBgn0039647	jus
FBgn0040257	Ugt302E1
FBgn0036844	Mkp3
FBgn0015791	Rab14
FBgn0015268	Nap1
FBgn0050361	mtt
FBgn0014163	fax
FBgn0029878	Pat1
FBgn0030037	Miga
FBgn0001233	Hsp83
FBgn0041605	cpx
FBgn0000412	D1
FBgn0011206	bol
FBgn0035638	Tektin-C
FBgn0005536	Mbs
FBgn0032378	CycY
FBgn0035107	mri
FBgn0025702	Srpk79D

FBgn0025382	Rab27
FBgn0034611	MFS16
FBgn0013275	Hsp70Aa
FBgn0002719	Men
FBgn0027865	Tsp96F
FBgn0028688	Rpn7
FBgn0266136	Gyc76C
FBgn0026064	KP78a
FBgn0037999	CG4860
FBgn0011211	blw
FBgn0000319	Chc
FBgn0027903	CG12018
FBgn0259108	futsch
FBgn0001234	lncRNA:Hsromega
FBgn0039109	CG10365
FBgn0250789	alpha-Spec
FBgn0037141	DNApol-eta
FBgn0266669	Sec3
FBgn0002284	Prosbeta6
FBgn0010309	pigeon
FBgn0010100	mAcon1
FBgn0286222	Fum1
FBgn0264816	koko
FBgn0264751	Vti1b
FBgn0023097	bon
FBgn0000546	EcR
FBgn0003435	sm
FBgn0043012	AP-2sigma
FBgn0041342	Pcyt1
FBgn0026376	Rgl
FBgn0004449	Ten-m
FBgn0087008	e(y)3
FBgn0004370	Ptp10D
FBgn0286813	SRPK
FBgn0264442	ab
FBgn0033246	ACC
FBgn0250843	Prosalpha6
FBgn0016003	l(3)61Da

FBgn0019644	ATPsynB
FBgn0011584	Trp1
FBgn0011224	heph
FBgn0002924	ncd
FBgn0025595	AkhR
FBgn0020493	Dad
FBgn0039738	Mgat2
FBgn0028622	qsm
FBgn0028567	robl62A
FBgn0023407	B4
FBgn0021760	chb
FBgn0004656	fs(1)h
FBgn0010113	hdc
FBgn0035965	Use1
FBgn0000448	Hr3
FBgn0000117	arm
FBgn0027084	LysRS
FBgn0027087	HisRS
FBgn0014010	Rab5
FBgn0010348	Arf79F
FBgn0010226	GstS1
FBgn0005640	Eip63E
FBgn0011573	Cdc37
FBgn0063493	GstE7
FBgn0037501	Ir84a
FBgn0005648	Pabp2
FBgn0037566	mRpL1
FBgn0020618	Rack1
FBgn0039271	CG11839
FBgn0003415	skd
FBgn0027654	jdp
FBgn0035423	eIF1
FBgn0266410	CG45050
FBgn0011760	ctp
FBgn0086655	jing
FBgn0040208	Kat60
FBgn0263601	mib1
FBgn0043884	mask
FBgn0023212	EloB

FBgn0015283	Rpn10
FBgn0037377	CG1218
FBgn0003935	snRNA:U5:34A
FBgn0003514	sqh
FBgn0028694	Rpn11
FBgn0262476	CG43066
FBgn0053100	eIF4EHP
FBgn0027055	CSN3
FBgn0000535	eag
FBgn0000405	CycB
FBgn0262601	SmB
FBgn0003008	or
FBgn0015622	Cnx99A
FBgn0027497	Madm
FBgn0003292	rt
FBgn0261954	east
FBgn0035827	Srp9
FBgn0259685	crb
FBgn0259246	brp
FBgn0053526	PNUTS
FBgn0261596	RpS24
FBgn0283521	lola
FBgn0038853	RhoGAP93B
FBgn0029155	Men-b
FBgn0000307	chif
FBgn0003044	Pcl
FBgn0266450	Kr-h1
FBgn0044414	Scim13
FBgn0030086	CCT2
FBgn0005355	Su(fu)
FBgn0010762	simj
FBgn0024314	Plap
FBgn0013751	Awh
FBgn0000256	capu
FBgn0020622	Pi3K21B
FBgn0259170	alpha-Man-Ia
FBgn0033313	Cirl
FBgn0278604	dmt
FBgn0016697	Prosalpha5

FBgn0261574	kug
FBgn0001989	ND-B17
FBgn0263108	BtbVII
FBgn0035023	ITP
FBgn0284436	CCT8
FBgn0025678	CaBP1
FBgn0035533	Cip4
FBgn0001994	crp
FBgn0011674	insc
FBgn0285896	btl
FBgn0067779	dbr
FBgn0020248	stet
FBgn0002891	mus205
FBgn0039234	Nct
FBgn0004839	otk
FBgn0261789	SmD2
FBgn0262511	Vha44
FBgn0068023	Dyak\Tal
FBgn0266848	wap
FBgn0000229	bsk
FBgn0040319	Gclc
FBgn0000318	cl
FBgn0265630	sno
FBgn0039581	Moca-cyp
FBgn0026375	RhoGAPp190
FBgn0028687	Rpt1
FBgn0003517	sta
FBgn0044415	Scim12
FBgn0003423	slgA
FBgn0032725	Nedd8
FBgn0261278	grp
FBgn0036688	Fit2
FBgn0003390	shf
FBgn0286070	cnk
FBgn0032216	Usp14
FBgn0261397	didum
FBgn0005624	Psc
FBgn0004569	aos
FBgn0001104	Galphai

FBgn0019936	RpS20
FBgn0022382	Pka-R2
FBgn0035936	Tsp66E
FBgn0023216	Parg
FBgn0032444	CCT4
FBgn0262126	Sec24CD
FBgn0033259	CG11210
FBgn0038320	Sra-1
FBgn0011754	PhKgamma
FBgn0029672	CG2875
FBgn0041102	ocn
FBgn0011766	E2f1
FBgn0041171	ago
FBgn0011818	oaf
FBgn0030365	Tango4
FBgn0028695	Rpn1
FBgn0022029	l(2)k01209
FBgn0032147	IP3K1
FBgn0264607	CaMKII
FBgn0029995	CG2256
FBgn0039172	Spase22-23
FBgn0266284	Ns3
FBgn0250823	gish
FBgn0011474	PR-Set7
FBgn0015245	Hsp60A
FBgn0003124	polo
FBgn0010830	l(3)04053
FBgn0024196	robl
FBgn0011260	Sema2a
FBgn0013718	nuf
FBgn0261822	Bsg
FBgn0031639	mRpS2
FBgn0011481	Ssdp
FBgn0024330	MED6
FBgn0005655	PCNA
FBgn0010470	Fkbp14
FBgn0003404	Si
FBgn0261445	sgl
FBgn0000404	CycA

FBgn0061515	endos
FBgn0027335	Rip11
FBgn0026417	Hus1-like
FBgn0032439	Ref2
FBgn0053202	dpr11
FBgn0261609	eIF2alpha
FBgn0034915	eIF6
FBgn0035534	mRpS6
FBgn0086355	Tpi
FBgn0260972	alc
FBgn0016034	mael
FBgn0085434	NaCP60E
FBgn0016032	lbm
FBgn0000308	chic
FBgn0033741	CG8545
FBgn0040395	Unc-76
FBgn0266098	rg
FBgn0039654	Brd8
FBgn0003206	Ras64B
FBgn0010220	Dbp45A
FBgn0003371	sgg
FBgn0262872	milt
FBgn0014179	gcm
FBgn0035150	Rev1
FBgn0263750	CG43675
FBgn0003118	pnt
FBgn0003175	px
FBgn0036030	CG6767
FBgn0027088	GlyRS
FBgn0039726	eIF2Balpha
FBgn0015799	Rbf
FBgn0023535	arg
FBgn0086358	Tab2
FBgn0028965	A16
FBgn0260400	elav
FBgn0043070	MESK2
FBgn0051155	Rpb7
FBgn0029134	Prosbeta5
FBgn0029820	CG16721

FBgn0028400	Syt4
FBgn0001169	H
FBgn0017418	ari-1
FBgn0025809	Paf-AHalpha
FBgn0010051	Itp-r83A
FBgn0036921	RhoGDI
FBgn0000273	Pka-C1
FBgn0259982	Uxt
FBgn0033494	KCNQ
FBgn0030067	Rbm13
FBgn0033379	Mys45A
FBgn0024251	bbx
FBgn0261608	RpL37A
FBgn0086707	ncm
FBgn0265487	mbl
FBgn0004390	RasGAP1
FBgn0001078	ftz-f1
FBgn0027360	Tim10
FBgn0266129	lov
FBgn0264078	Flo2
FBgn0035063	CG3594
FBgn0020496	CtBP
FBgn0261238	Alh
FBgn0040071	tara
FBgn0037351	RpL13A
FBgn0035246	CG13928
FBgn0035473	mge
FBgn0264816	koko
FBgn0038271	UQCR-C1
FBgn0033555	RpS15Ab
FBgn0039626	Slu7
FBgn0020386	Pdk1
FBgn0039632	Cul5
FBgn0014269	prod
FBgn0039006	Cyp6d4
FBgn0261592	RpS6
FBgn0001225	Hsp26
FBgn0011725	twin
FBgn0014133	bif

FBgn0260635	Diap1
FBgn0285950	RpL19
FBgn0032680	Ntf-2r
FBgn0262519	Mi-2
FBgn0069242	eca
FBgn0026380	Prosbeta3
FBgn0023174	Prosbeta2
FBgn0039116	CG10375
FBgn0028331	l(1)G0289
FBgn0033378	tsu
FBgn0037543	CG10903
FBgn0267791	HnRNP-K
FBgn0010228	HmgZ
FBgn0003134	Pp1alpha-96A
FBgn0283473	S6KL
FBgn0043841	vir-1
FBgn0001316	klar
FBgn0035047	Pof
FBgn0266377	Pde8
FBgn0035978	UGP
FBgn0031062	CG14230
FBgn0010415	Sdc
FBgn0023091	dimm
FBgn0265523	Smr
FBgn0000152	Axs
FBgn0013733	shot
FBgn0023549	Mct1
FBgn0004838	Hrb27C
FBgn0014388	sty
FBgn0028507	CG3793
FBgn0035422	RpL28
FBgn0030242	sofe
FBgn0285962	CG46338
FBgn0003884	alphaTub84B
FBgn0020369	Rpt6
FBgn0039139	Mettl3
FBgn0261985	Ptpmeg
FBgn0030766	mthl1
FBgn0016131	Cdk4

FBgn0261239	Hr39
FBgn0265523	Smr
FBgn0262614	pyd
FBgn0011708	Syx5
FBgn0028689	Rpn6
FBgn0041184	Socs36E
